# Acceptance and completion of rifapentine-based TB preventive therapy (3HP) among people living with HIV (PLHIV) in Kampala, Uganda—patient and health worker perspectives

**DOI:** 10.1186/s43058-021-00173-2

**Published:** 2021-06-30

**Authors:** Fred C. Semitala, Allan Musinguzi, Jackie Ssemata, Fred Welishe, Juliet Nabunje, Jillian L. Kadota, Christopher A. Berger, Achilles Katamba, Noah Kiwanuka, Moses R. Kamya, David Dowdy, Adithya Cattamanchi, Anne R. Katahoire

**Affiliations:** 1 Department of Medicine, School of Medicine, Makerere University College of Health Sciences, Kampala, Uganda; 2 Makerere University Joint AIDS Program (MJAP), Kampala, Uganda; 3 Infectious Diseases Research Collaboration, Kampala, Uganda; 4 Division of Pulmonary and Critical Care Medicine and Center for Tuberculosis, University of California San Francisco, San Francisco, CA USA; 5 Uganda Tuberculosis Implementation Research Consortium, Kampala, Uganda; 6 School of Public Health, Makerere University College of Health Sciences, Kampala, Uganda; 7 Department of Epidemiology, Johns Hopkins Bloomberg School of Public Health, Baltimore, MD USA; 8 Center for Vulnerable Populations at Zuckerberg San Francisco General Hospital and Trauma Center, University of California, San Francisco, San Francisco, CA USA; 9 Child Health and Development Centre, Makerere University College of Health Sciences, Kampala, Uganda

**Keywords:** Tuberculosis, Preventive therapy, Perceived barriers, Perceived facilitators, People living with HIV, Health workers, Implementation, Qualitative methods

## Abstract

**Background:**

A 12-dose, once-weekly regimen of isoniazid and rifapentine (3HP) is effective in preventing tuberculosis (TB) among people living with HIV (PLHIV). We sought to identify potential barriers to and facilitators of acceptance and completion of 3HP treatment from the perspective of people living with HIV (PLHIV) and health workers in a routine HIV care setting in Kampala, Uganda.

**Methods:**

We conducted semi-structured interviews with 25 PLHIV and 10 health workers at an HIV/AIDS clinic in Kampala, Uganda. For both groups, we explored their understanding and interpretations of TB and TB preventive therapy (TPT), and perceptions about social and contextual factors that might influence the willingness of PLHIV to initiate and complete 3HP. We analyzed the data using an inductive thematic approach and aligned the emergent themes to the Behavior Change Wheel framework to identify sources of behavior and targeted behavior change interventions.

**Results:**

Facilitators of acceptance and completion of 3HP treatment among PLHIV were fear of contracting TB, awareness of being at risk of getting TB, willingness to take TPT, trust in health workers, and the perceived benefits of directly observed therapy (DOT) and self-administered therapy (SAT) 3HP delivery strategies. Barriers included inadequate understanding of TPT, fear of potential side effects, concerns about the effectiveness of 3HP, and the perceived challenges of DOT or SAT. Among health workers, perceived facilitators included knowledge that TB is a common cause of mortality for PLHIV, fear of getting TB, and trust in the health workers by PLHIV, the advantages of once-weekly 3HP dosing, and the benefits of DOT and SAT 3HP delivery strategies. Health worker-reported barriers for PLHIV included inadequate understanding of TB and benefits of TPT, TB-associated stigma, potential side effects pill burden, and challenges of DOT and SAT 3HP delivery strategies. Lack of experience in the use of digital technology to monitor patient care was identified as a health worker-specific barrier. Identified intervention functions to address the facilitators or barriers included education, persuasion, environmental restructuring, enablement, and training.

**Conclusions:**

Using a formative qualitative and comprehensive theoretical approach, we identified key barriers, facilitators, and appropriate interventions, including patient education, enhancing trust, and patient-centered treatment support that could be used to optimize the delivery of 3HP to PLHIV in our setting. These interventions are likely generalizable to other clinical interventions in similar populations in sub-Saharan Africa and other TB high-burden settings.

**Supplementary Information:**

The online version contains supplementary material available at 10.1186/s43058-021-00173-2.

Contributions to the literature

- The innovation in this study is the application of an implementation science-informed approach to an important global health problem: scale-up of short-course preventive therapy for tuberculosis, a leading cause of morbidity and mortality.
- We identify and categorize key barriers to scale up in a manner that can allow others to verify similar barriers in their settings and inform intervention development and testing.

## Background

Tuberculosis (TB) is the leading cause of death among people living with HIV (PLHIV) worldwide [1]. TB preventive therapy (TPT) is recommended by the World Health Organization (WHO) for all PLHIV without active TB [2–4]. The traditional TPT regimen has involved PLHIV taking 6 to 9 months of daily isoniazid [5]. However, acceptance and completion of daily isoniazid treatment among PLHIV have been poor worldwide due to concerns about toxicity and the long duration of treatment [6, 7].

As an alternative, in 2018, WHO recommended a 3-month (12-dose) regimen of weekly isoniazid and rifapentine (3HP) based on randomized trials showing equivalent efficacy, better tolerability, and higher treatment completion compared to 6 months of daily isoniazid [4, 8, 9]. Sterling et al. compared the efficacy of 3HP to 9 months of daily isoniazid monotherapy (9H) in the prevention of active TB disease among people at high risk of developing TB disease in the USA, Canada, Brazil, and Spain. 3986 participants were randomized to the 3HP arm compared to 3745 in the 9H arm [8]. In a randomized trial, Martinson et al. evaluated the efficacy of 3HP (328 participants) compared to 6 months of isoniazid monotherapy (328 participants) among HIV-positive adults in Soweto, South Africa [9]. In both studies, each weekly dose of 3HP was administered to PLHIV under direct observation of a health worker (directly observed therapy, DOT) to ensure adherence and provide an opportunity for monitoring and managing adverse events. Participants were considered to have completed their 3HP treatment if they were observed taking at least 90% (11 of the 12 doses) of their assigned doses by a health worker. 3HP treatment completion rates of up to 95% [9] compared to 83.8% of isoniazid monotherapy [9] were observed using DOT. However, DOT may pose challenges to the acceptance and completion of 3HP among PLHIV and health workers in low-income countries. These challenges include direct and indirect costs of attending weekly clinic visits consuming up to 40% of median personal weekly income [10], lack of time to attend clinic visits, and increased workload for health workers. While self-administered therapy (SAT) overcomes some of these challenges, it could lead to delayed recognition of adverse events and lower treatment completion in some contexts [11].

Formative studies help researchers to optimize the design and implementation of evidence-based interventions by tailoring them to fit within the real-world practice contexts of key stakeholders [12–15]. Fostering behavior change, underpinned by a theoretical understanding of the target behaviors among stakeholders, including patients and health workers, is key to improving healthcare and health outcomes [16–18].

The Capability, Opportunity, Motivation, Behavior (COM-B) model is a theory of behavior that can help us understand TPT acceptance and completion among PLHIV in Uganda. The COM-B model proposes that changing the behavior of an individual, a group or a population requires changing either their capability, opportunity, and/or motivation needed to perform the behavior [19, 20].

Capability can be psychological (knowledge and psychological skills) or physical (physical strength and/or skills); opportunity can be physical (environmental factors such as time, resources, location) or social (social cues, interpersonal relationships, and cultural norms); motivation can be automatic (emotions, impulses, needs, and wants) or reflective (self-conscious planning and evaluations/beliefs). Based on its multiple, distinct explanatory components, and their underlying domains, the COM-B model offers the opportunity to explore several potential influences on behavior [18].

COM-B lies at the center of the Behavior Change Wheel (BCW). The BCW is a theory and evidence-based tool that provides a systematic method for identifying effective intervention functions to target facilitators or barriers within each COM-B domain. Thus, the BCW framework provides a coherent basis for considering potential barriers and facilitators to behavior change and the interventions expected to overcome those barriers or promote identified facilitators in a given context [21].

The aim of this qualitative study was to identify potential barriers to, and facilitators of, 3HP acceptance and completion, from the perspective of PLHIV in HIV/AIDS care and health workers at a large, urban HIV/AIDS clinic in Kampala, Uganda, and map these onto the COM-B model and BCW framework to identify suitable intervention functions. The goal was to inform the design of SAT and DOT delivery strategies for a type 3 hybrid effectiveness-implementation trial of 3HP [22].

## Methods

### Study design and population sample

We conducted semi-structured interviews between January and March 2019 among PLHIV and health workers at the Mulago Immune Suppressive Syndrome (i.e., AIDS) clinic in Kampala, Uganda. The Mulago AIDS clinic provides comprehensive HIV/TB care and treatment services to over 16,000 PLHIV, largely from Kampala, the capital city of Uganda, and surrounding districts. We purposively sampled consecutive PLHIV engaged in care at the clinic during their routine appointments. A sample size of 20 PLHIV was predetermined as a minimum guided by Green and Thorogood [23]. However, the maximum sample of PLHIV was determined by data saturation as proposed by Lincoln and Guba [24]. We also purposively sampled all full-time health workers who were involved in the provision of TB treatment and prevention services at the clinic. Interviews with PLHIV and health workers lasted between 25 and 40 min. Informed verbal consent was obtained from all participants.

The study received ethical approvals from the School of Public Health Higher Degrees Research and Ethics Committee at the Makerere University College of Health Sciences, the Uganda National Council of Science and Technology, and the University of California San Francisco Committee on Human Research. We used the Standards for Reporting Qualitative Research (SRQR) and the Consolidated Criteria for Reporting Qualitative Research (COREQ) checklists in the reporting of this qualitative study (see Additional file 1).

### Recruitment of study participants

All adult (≥18 years old) PLHIV engaged in care at the clinic who were able to provide informed verbal consent in English or Luganda (the most widely spoken local language) were considered for enrollment into the study. PLHIV were recruited by two study nurses from the patient waiting area or were referred by clinic staff at the patient triage area. The study team purposively sampled PLHIV to achieve variation based on the duration of care at the clinic, as well as demographic characteristics such as sex, age, and distance traveled to reach the clinic. Health workers were recruited based on their active involvement in providing TB-related care at the clinic. All participants were informed that the goal of this research was to inform the design of an implementation trial of 3HP that was going to be conducted at the same clinic.

### Study instruments and data collection

Separate interview guides were developed for PLHIV and health workers. Both interview guides included open-ended questions framed using the COM-B model, designed to explore potential barriers to and facilitators of acceptance and completion of 3HP treatment offered via DOT or SAT delivery strategies by PLHIV at the Mulago AIDS clinic. We defined acceptance of 3HP as the willingness by PLHIV to take the once-weekly dose of 3HP prescribed via DOT/SAT by health workers at the clinic. We defined completion of 3HP treatment as PLHIV swallowing at least 11 of 12 once-weekly doses within 16 weeks of enrollment.

The interview guides for PLHIV explored their understanding of TB (i.e., the disease itself, how it is transmitted, its prevention and treatment), their perceptions of individual risk of contracting TB disease and awareness about TPT, and their perception of its importance. Other questions were specific to 3HP and explored willingness to take 3HP if it were offered to them by their health worker, how easy/difficult it would be to remember to take the once-weekly dose, perceived advantages or disadvantages of either taking 3HP via DOT or SAT, and perceptions about the use of digital adherence technologies to support 3HP adherence and completion.

The interview guides for health workers included questions about their experiences with PLHIV at the clinic, their experiences with TB and TPT, and their perceptions about concerns that PLHIV may have about TB and TPT. The guide also explored perceived challenges or benefits that their clients may encounter with DOT vs. SAT, their own preferences for delivering 3HP by DOT vs. SAT, and their perceptions about the use of digital adherence technologies to support 3HP completion.

Both interview guides were drafted in English, piloted, and refined using a convenience sample of PLHIV and health workers at the clinic. The interview guide for PLHIV was then professionally translated to Luganda and checked for accuracy by members of the local study team who were bilingual.

All interviews including the informed consent were audio-recorded and transcribed verbatim. All Luganda transcripts were then translated to English for analysis. All transcripts were de-identified and stored in a secure digital folder accessible only by the research team.

### Research team

All interviews were conducted at the Mulago AIDS Clinic, a setting familiar to study participants, by the local study team. The team comprised of social scientists and several experienced TB/HIV researchers who had previously conducted TB research together [25–27]. A doctoral trained social scientist, employed as a senior lecturer at Makerere University, Kampala (ARK, female), trained the team before the data collection and supervised the data collection process. A bachelor’s trained social scientist (JS, female) conducted the initial interviews with PLHIV while the two study nurses (FW, male; JN, female) and a medical doctor (AM, male) attended the sessions to take notes. Subsequent interviews with PLHIV were conducted by the social scientist and the study nurses. All interviews with health workers were conducted by the medical doctor and study nurses. The interviewers did not know the study participants prior to study commencement.

### Analysis

Data was analyzed by three members of the research team (FS, AM, ARK) using a thematic approach. Thematic analysis was preferred because it is suitable for examining the perspectives of different research participants, highlighting similarities and differences, and generating unanticipated insights [28–30]. An inductive approach was adopted using open coding that facilitated the identifying of themes in the data. Initially, two analysts (AM, ARK) read three similar transcripts independently familiarizing themselves with the data and documenting thoughts on potential codes and themes. The team then met to debrief and compare the initial codes generated by each analyst. Coding discrepancies were discussed and resolved with a third member of the research team (FS). A coding framework was then developed and applied to the remaining transcripts. The themes emerging during the coding processes were noted and reviewed in subsequent team meetings where they were discussed and a consensus on the themes documented. Investigator triangulation and peer debriefing were maintained throughout the analysis process including in the defining and naming of themes.

The emergent themes were then categorized as either potential facilitators or barriers to the acceptance and completion of 3HP treatment by PLHIV. Potential barriers were defined as perceived constraints to the acceptance and completion of 3HP treatment by PLHIV. Potential facilitators were defined as potential enablers of acceptance and completion of 3HP treatment by PLHIV. We then mapped the emergent barriers and facilitators onto their associated sources of behavior on the COM-B model, to develop a behavioral diagnosis [19]. Finally, we used the BCW framework [28] to identify potential interventions to overcome the barriers and promote the facilitators of acceptance and completion of 3HP by PLHIV.

## Results

### Demographic characteristics of study participants

Twenty-five (25) PLHIV and 10 health workers participated in the interviews. Of the 25 PLHIV, 16 (64%) were female, and the median age was 39 years (IQR: 31–45). Their duration in HIV/AIDS care at the clinic ranged from 8 months to 14 years (median 6 years; IQR: 5–10 years). The 10 health workers included three medical doctors, three nurses, two clinical officers, one pharmacist, and one pharmacy technician. Six health workers (60%) were female, and the median age was 29.5 years (IQR: 28–35 years). The duration in service at the current post ranged from 6 months to 11 years (median 2.5 years; IQR: 2–7 years).

### Patient-reported facilitators

Almost all PLHIV were aware that their HIV-positive status put them at a higher risk of developing active TB and were willing to take 3HP to lower their risk. They also understood that TB can easily spread from person to person. Almost all were open to taking medicines if it was recommended by their health workers at the clinic in order to reduce their chances of contracting TB. Table 1 summarizes the patient-reported facilitators and barriers to acceptance, completion, and models of delivery of 3HP treatment.

“TB is a terrible disease; what I know about it is it spoils one of the organs and that is the lungs and once the lungs are spoilt then of course next is death……And I have seen very many people suffering from TB you wouldn’t like the same. So, if there is a chance of prevention, I welcome it”. (Middle-aged Male, PLHIV interview)

**Table 1:** Patient-reported barriers to and facilitators of the use, completion, and models of delivery for 3HP at Mulago AIDS clinic in Kampala, Uganda

Potential facilitators	Potential barriers
**Fear of contracting TB -** *TB is a terrible disease what I know about it is it spoils one of the organs and that is the lungs and once the lungs are spoilt then of course next is death……And I have seen very many people suffering from TB you wouldn’t like the same. So, if there is a chance of prevention, I welcome it. (Middle-aged Male, PLHIV interview)*	**Inadequate understanding of TPT -** *My thoughts are why do you give me medicine if I am healthy…. You may find that God did not plan for them to die of the disease-causing organism for TB and yet they are taking medicine… No. I do not support that! (Young Female, PLHIV interview)*
**Aware of being potentially at risk of TB -** *Yes, mostly people who have HIV it is so good because our bodies are not strong it gets sick easily but if I prevent it means it is not easy to get. (Young Female, PLHIV interview)*	**Potential pill burden** ***-*** *The problem is because for me I have been taking 2 tablets (Septrin and ARVs), now what has shocked me is taking 11 tablets. I don’t know the danger with taking because I have never taken such many tablets. (Middle-aged Male, PLHIV interview)*
**Awareness that TB is easily transmissible -** *What I know is that when someone is suffering from it, there is a high chance when you are sitting with that one, when you are sharing cups, when she is a wife and you are sleeping together, when you are a parent and your children are there, of course there is a lot of communication. So, it is terrible if you have a home, the whole home may be affected. (Middle-aged Male, PLHIV interview)*	**Fear of potential side effects** ***-*** *The question I can ask about is whether it can have side effects. Those side effects are the ones we fear the most about medicine because now we see prevention but if I started it what would I look like? What would it make me look like? How would it treat me? All those things have to be known because you can start taking it and you become very strange. The eyes change, and you get to a point where you cannot do your work to earn a living. Yet also if you are taking medicine but are not feeding well you may not stay long. (Young Female, PLHIV interview)*
**Willingness to take TB preventive therapy -** *Eeh! My dear, they say prevention is better than cure. That is why I am here. You never know you can get that disease and you get problems treating it. But if you prevent against it and you do not get it, it’s better. (Middle-aged Male, PLHIV interview)*	**Concerns about the effectiveness of 3HP -** *My concern might be, leave alone the side effects when you take it aren’t you very vulnerable to contract TB again…. So, I don’t know, will I be vulnerable to such TB or I will be protected? (Middle-aged Female, PLHIV interview)*
**Trust in health workers -** *No, I don’t see any problem what I have realized with experience is that the medical people can’t recommend something that can be harmful. (Middle-aged Male, PLHIV interview)*	**Perceived challenges of DOT** ***-*** *One challenge would be transport costs will be high…. Transport costs, sometimes time, and maybe getting permission from work. (Young Female, PLHIV interview)*
**Perceived benefits of DOT -** *Because they take under your observation and you make sure that they have taken it. You know that they have taken it but this person taking from home is on probability. They may take or may not take, and you would not know. But for the person here you would be on sure deal because you have observed them taking it. (Middle-aged Female, PLHIV interview)*	**Perceived challenges of SAT** ***-*** *Now doctor, some people you can give them medicine and when they come here, and you ask them; did you take your medicine? They will say yes. But truthfully, they got 11 tablets, took three of them and stopped. And yet, you told them, you take all 11 tablets at once. They will take only two tablets and the rest, they fail. If they get time like the following day, then they take again. Doesn’t that spoil it? I think it spoils it! (Middle-aged Female, PLHIV interview)*
**Perceived benefits of SAT** ***-*** *You are not inconvenienced. You can even take the medicine and finish it without anyone knowing that you are on medication. Even at work you will not be disturbed by having to seek permission. (Middle-aged Female, PLHIV interview)*	

*Abbreviations*: *3HP* Rifapentine-Isoniazid combination, *TB* Tuberculosis, *PLHIV* Person/people living with HIV, *TPT* Tuberculosis preventive therapy, *HIV* Human immunodeficiency virus, *ARV* Antiretroviral drug, *DOT* Directly observed therapy, *SAT* Self-administered therapy

*Septrin*: antibiotic combination of trimethoprim/sulfamethoxazole

Most PLHIV preferred self-administration of 3HP as it would be cheaper, would suit their daily lives better, and would allow them to take their medicines at their convenience.

You are not inconvenienced. You can even take the medicine and finish it without anyone knowing that you are on medication. Even at work you will not be disturbed by having to seek permission. (Middle-aged Female, PLHIV interview)

At the same time, the delivery of 3HP using the DOT strategy was perceived by a few PLHIV as potentially beneficial given greater contact with the health workers, who would ensure that they took the medication and could address any patient concerns or side effects.

### Patient-reported barriers

Several of the PLHIV interviewed were (1) unaware that TB could be prevented, (2) did not understand the importance of TB preventive therapy, or (3) queried the logic of treating someone who is not “sick” with TB:

My thoughts are why you give me medicine if I am healthy…. You may find that God did not plan for them to die of the disease-causing organism for TB and yet they are taking medicine… No. I do not support that! (Young Female, PLHIV interview)

PLHIV perceived the weekly 3HP dose, which consists of 11 tablets, as too many and potentially prohibitive. Some doubted their own ability to swallow 11 tablets at once:

The problem is because for me I have been taking 2 tablets [Septrin and ARVs], now what has shocked me is taking 11 tablets. I don’t know the danger with taking because I have never taken such many tablets. (Middle-aged Male, PLHIV interview)

PLHIV feared potential side effects, believing that the medicines used to treat TB were “tough” on the body and were concerned that they could arise especially when combined with their current ART regimens. Some of the participants were also concerned about the duration of protection afforded by 3HP:

My concern might be, leave alone the side effects when you take it aren’t you very vulnerable to contract TB again…. So, I don’t know, will I be vulnerable to such TB or will I be protected? (Middle-aged Female, PLHIV interview).

The weekly clinic appointments associated with DOT were perceived as costly in terms of transport, time, childcare, and absence from work. Those who resided far away from the clinic expressed additional concern:

One challenge would be transport costs will be high…. Transport costs, sometimes time, and maybe getting permission from work. (Young Female, PLHIV interview)

SAT as a delivery strategy was perceived to be convenient, but some PLHIV expressed that the lack of dosing supervision combined with the unusual once-weekly dosing schedule could potentially lead to poor adherence to 3HP:

Now doctor, some people you can give them medicine and when they come here, and you ask them; did you take your medicine? They will say yes. But truthfully, they got 11 tablets, took three of them and stopped. (Middle-aged Female, PLHIV interview)

### Health worker-reported facilitators

All health workers interviewed mentioned that TB was the leading cause of severe illness and death among PLHIV in care at the clinic and perceived TB prevention as very important. Table 2 summarizes the health worker-reported facilitators and barriers to acceptance, completion, and models of delivery of 3HP treatment.

In this clinic, of the 16,000 patients, most are stable and if you look at the cause of mortality it is TB related. … So, it deserves much attention. (Medical Doctor at the clinic)

**Table 2:** Health worker-reported barriers to and facilitators of the use, completion, and models of delivery for 3HP at Mulago AIDS clinic in Kampala, Uganda

Potential facilitators	Potential barriers
***Common cause of mortality among stable PLHIV -*** *In this clinic, of the 16,000 patients, most are stable and if you look at the cause of mortality it is TB related. … So, it deserves much attention. (Medical Doctor at the clinic)*	**Inadequate understanding of TB and TPT** ***-*** *Some people think that TB is in a family, that it’s hereditary. So, they will tell you, “You know my father had TB, so I also have TB or me I can’t have TB in our family, no one has ever had TB”. Such people will not adhere well on treatment. (Nurse at the clinic)*
**PLHIV trust their health workers** ***-*** *If you explain to them the benefits of taking this preventive treatment, they are usually receptive, and they usually take health workers information as kind of gospel truth. (Medical Doctor at the clinic)*	**Stigma associated with TB** ***-*** *We get challenges in trying to implement infection control procedures here because of the stigma associated with it even in the clinic. (Nurse at the clinic)*
**Fear of TB*-*** *They think ...someone may die if they get the disease. They really have a great fear of the disease…. when they hear that there are preventive measures, everyone will want to take it to prevent them from not getting TB. (Nurse at the clinic)*	**Fear of potential side effects** - *“Balese biragala kututta.” (this literally means they have brought medications to kill us) … And then other people think that the TB prevention medications, are very strong, they are very toxic that they will affect the liver and the kidney. (Nurse at the clinic)*
**Receptiveness to TPT** ***-*** *They seem to be eager to take the medicine including some requesting for it even though it was not prescribed for them …. They would say how come for me I haven’t received? ….… So, when they hear of the preventive treatment, they will not hesitate to start the drugs. (Pharmacy Technician at the clinic)*	**Potential pill burden** ***-*** *Musawo (meaning health worker in the local language), I have been concentrating on my triple (three daily ARV pills) and now you want to give me more medication. …some of them are not so positive about it. (Medical Doctor at the clinic)*
**Once-weekly 3HP dosing schedule -** *Taking it once a week rather than taking it daily much as the tablets are many. Maybe one will be like “anyway am taking many but once a week”. (Medical Doctor at the clinic)*	**Perceived challenges of DOT** ***-*** *Their work schedule I don’t think it would allow them to come weekly. ….. most of our clients they are not self-employed, they are employed, and they have not disclosed……. So, it’s very hard for someone to ask for permission every week the boss will get suspicious…. others travel long distances; others travel a lot. (Nurse at the clinic)*
***Streamlined clinic visits -*** *If there is one specific staff, let me say nurse giving out this medicine it will help the patient and the clinic……… the patient will know where to go and not meander around. And even for the health workers, it will maximize the patient flow and the congestion in the clinic. (Nurse at the clinic)*	**Perceived challenges of SAT -** *In DOT they take the medicine when you are really seeing, you can be sure that they are taking it. But for those ones taking it from home, sometimes they can take less, and you can’t know. (Nurse at the clinic)*
**Perceived benefits of DOT** ***-*** *First, when this person comes weekly, the health worker will be able to see if there is any change; early detection of side effects that is one. And then second you are going to be sure this person has taken his or her medication. Because you are going to be there and see. (Nurse at the clinic)*	**Technology challenges -** *Technology no, mostly we depend on self-report, pill count, say I have been taking so what is your balance? Then you negotiate around that. Technology no. (Nurse at the clinic)*
**Perceived benefits of SAT** ***-*** *First, the time you spend coming to the hospital, you save that time and transport. You would be doing some other things instead of coming to the hospital. And this person secures his or her job, yes. (Nurse at the clinic)*	

*Abbreviations*: *3HP* Rifapentine-isoniazid combination, *TB* Tuberculosis, *PLHIV* Person/people living with HIV, *TPT* Tuberculosis preventive therapy, *ARV* Antiretroviral drug, *DOT* Directly observed therapy, *SAT* Self-administered therapy

Health workers believed that the fear of getting TB would motivate PLHIV to take and complete 3HP. They also conceded that the once-weekly dosing schedule of 3HP could convince some PLHIV to take it despite the potential pill burden.

Taking it once a week rather than taking it daily much as the tablets are many. Maybe one will be like “anyway am taking many but once a week”. (Medical Doctor at the clinic)

Most health workers preferred to offer 3HP to PLHIV using DOT to ascertain completion of the required doses but acknowledged that most PLHIV in their care would most likely prefer SAT given the implications of costs and convenience. Conflicting work schedules were also perceived as a potential challenge for weekly clinic appointments.

### Health worker-reported barriers

Almost all the health workers felt that taking 11 tablets at once would be perceived as too high a pill burden, especially for those already struggling to adhere to their daily ART.

…I have been concentrating on my triple [three daily ARV pills] and now you want to give me more medication. …some of them are not so positive about it. (Medical Doctor at the clinic).

Health workers perceived inadequate knowledge about TB as a potential challenge for PLHIV to accept and complete 3HP. They noted that while most PLHIV knew the common signs and symptoms of TB, they still had misconceptions such as TB being hereditary, or transmitted through cigarette smoking and alcohol drinking. These misconceptions could potentially result in non-acceptance or non-completion of TPT once initiated on treatment. They also noted that TB is a highly stigmatized disease both at the clinic and within the communities where patients reside. Due to this and drawing from their experiences at the clinic, some health workers believed that PLHIV would be uncomfortable to be seen taking TB medicines. Health workers also observed that PLHIV would be concerned about potential side effects, while others mistrusted any new medications:

“Balese biragala kututta.” (This literally means they have brought medications to kill us) … “And then other people think that the TB prevention medications, are very strong, they are very toxic that they will affect the liver and the kidney”. (Nurse at the clinic)

Finally, most health workers reported that they had never used technology to monitor drug adherence of PLHIV remotely and were uncertain about the reliability of adherence determined electronically:

Technology no, mostly we depend on self-report, pill count, say I have been taking so what is your balance? Then you negotiate around that. Technology no. (Nurse at the clinic)

### Behavioral diagnosis and intervention options

Using the COM-B model, we categorized the barriers and facilitators to acceptance and completion of 3HP treatment reported by both PLHIV and health workers into their behavioral determinants and thereby developed a “behavioral diagnosis” for the identified barriers and facilitators. For example, inadequate understanding of TB and TB preventive therapy was categorized as a psychological capability barrier using the COM-B model. Table 3 summarizes the PLHIV and health worker-reported facilitators and barriers expressed in terms of their behavioral determinants within the COM-B model.

**Table 3:** Perceived facilitators and barriers to acceptance and completion of 3HP expressed in terms of their Behavioral determinants within the COM-B model

Behavioral Determinants	Emergent themes	
	PLHIV	Health workers
**Facilitators**		
Capability (psychological)	• Aware of being potentially at risk of TB • Awareness of easy transmission of TB	• Knowledge that TB causes the highest mortality among PLHIV at the clinic
Opportunity (physical)		• Streamlined clinic visits
Motivation (reflective)	• Willingness to take TPT • Trust in health workers • Perceived benefits of DOT/SAT	• PLHIV trust health workers • PLHIV are receptive to TPT • Convenience of once-weekly 3HP dosing schedule • Perceived benefits of DOT/SAT
Motivation (automatic)	• Fear of contracting TB	• PLHIV fear TB
**Barriers**		
Capability (physical)	• Potential pill burden (difficulty to swallow many pills)	• Technology challenges • Potential pill burden (difficulty to swallow many pills)
Capability (psychological)	• Inadequate understanding of TPT	• Inadequate understanding of TB and TPT
Opportunity (social)		• Stigma associated with TB
Motivation (reflective)	• Perceived challenges of DOT/SAT • Concerns about the effectiveness of 3HP	• Perceived challenges of DOT/SAT
Motivation (automatic)	• Fear of potential side effects	• PLHIV’s fear of potential side effects

*Abbreviations*: *3HP* rifapentine-Isoniazid combination, *COM-B* capability opportunity motivation behavior model, *TB* tuberculosis, *PLHIV* person/people living with HIV, *TPT* tuberculosis preventive therapy, *DOT* directly observed therapy, *SAT* self-administered therapy

By linking the behavioral diagnosis obtained using the COM-B model to the BCW framework, we identified appropriate functions that potential interventions could serve to address the reported barriers and facilitators and thereby enhance the acceptance and completion of 3HP by PLHIV. Table 4 shows the investigator identified intervention functions that can target the reported barriers and facilitators. For example, PLHIV and health workers appreciated the importance of improving the knowledge of PLHIV about TB and TPT through *education*. The established trust between PLHIV and the health workers can be used to encourage acceptance of 3HP through *persuasion.*

**Table 4:** Investigator-identified intervention functions targeting identified barriers and facilitators as defined in the behavior change wheel framework

Intervention functions	Potential interventions
**Education**	1. Clinic to adopt regular TB/TPT health education talks for PLHIV
	2. Counselling PLHIV about TB and TPT prior to initiation of 3HP
**Persuasion**	1. Health workers to help convince PLHIV to take 3HP
	2. Leverage once-weekly dosing schedule to convince PLHIV to take 3HP
**Training**	1. Train health workers on how to use digital adherence technology
**Environmental restructuring**	1. Reduce waiting time for PLHIV by streamlining DOT clinic visits
**Enablement**	1. Weekly dosing reminders for PLHIV taking 3HP as SAT
	2. Consider using fixed-dose combination pills of 3HP to reduce pill burden
	3. Provide emotional support for PLHIV using counselors at the clinic

*Abbreviations*: *3HP* rifapentine-isoniazid combination, *TB* tuberculosis, *PLHIV* person/people living with HIV, *TPT* tuberculosis preventive therapy, *DOT* directly observed therapy, *SAT* self-administered therapy

On the other hand, the waiting time associated with 3HP clinic visits can be addressed through *environmental restructuring* interventions such as having a dedicated space in the clinic and designated clinic staff for TPT to streamline patient flow. Concerns about once-weekly dosing and absence of dosing supervision for 3HP administered through SAT can be addressed by *enablement*, via weekly dosing reminders to PLHIV. Finally, health workers can be empowered to remotely monitor 3HP adherence for PLHIV on SAT through *training* on the use of digital adherence monitoring technologies. Tables 5 and 6 provide a summary of the intervention functions selected by the investigators to promote the facilitators and address the barriers to 3HP acceptance and completion by PLHIV.

**Table 5:** Summary of identified facilitators and linked intervention functions

Capability			Opportunity	Motivation					Intervention functions
Psychological			Physical	Reflective				Automatic	
Aware of being at risk for TB	Aware of easy transmission of TB	Knowledge that TB causes the highest mortality	Streamlined clinic visits	Willingness to take TPT	Trust in health workers	Perceived benefits of DOT/SAT	Convenience of once-weekly 3HP dosing schedule	Fear of TB	
✓	✓	✓		✓	✓				Enablement
							✓	✓	Persuasion
			✓						Environmental restructuring

**Table 6:** Summary of identified modifiable barriers and selected linked intervention functions

Capability			Opportunity	Motivation			Intervention Functions
Psychological	Physical		Social	Reflective		Automatic	
Inadequate understanding of TB & TPT	Health worker technology challenges	Pill burden	Stigma	Concerns about the effectiveness of 3HP	Perceived challenges of DOT & SAT	Fear of potential side effects	
✓				✓			Education
		✓	✓		✓		Enablement
		✓		✓		✓	Persuasion
					✓		Environmental restructuring
	✓						Training

## Discussion

The goal of this formative qualitative study was to identify potential barriers to and facilitators of acceptance and completion of 3HP TPT, as perceived by PLHIV and health workers, to inform the design of optimized SAT and DOT delivery strategies. The key potential facilitators for acceptance and completion of 3HP among PLHIV included fear of contracting TB, trust in health workers, the once-weekly 3HP dosing schedule, and the perceived benefits of both DOT and SAT delivery strategies. Key potential barriers identified were inadequate understanding of TPT, fear of 3HP pill burden, potential side effects, concerns about the effectiveness of 3HP, and the perceived challenges of DOT or SAT delivery strategies. In addition, health workers also had no prior experience with the use of digital technology to monitor patient care. In summary, neither DOT or SAT are likely to work for all patients, suggesting that offering an informed choice between the two should also be considered as part of scale-up. These findings highlight the need for targeted implementation support to enhance uptake of 3HP in this setting.

Studies from other high HIV and TB burden countries have shown that ineffective communication between patients and health workers may lead to a misunderstanding of the preventive role of TPT by patients and influences their acceptance of and completion of TPT [31, 32]. Both the PLHIV and health workers affirmed that patients trust the health workers at the clinic and would be receptive to 3HP if their health worker recommended it. Once patients are convinced about the benefits and delivery strategies for 3HP, they are well-positioned to guide its scale-up. In addition, health workers believed that the once-weekly 3HP dosing schedule could be used as an advantage to encourage PLHIV to accept and complete treatment. Several studies have previously demonstrated that trust in health workers influences patient adherence to and completion of TPT [31–33].

Other perceived challenges associated with 3HP delivery included increased costs of attending more frequent clinic visits, conflicting work schedules, and forgetting to take 3HP dosages due to the unfamiliar once-weekly dosing schedule. Other studies have also reported clinic-based DOT, financial barriers, forgetfulness, work duties, childcare responsibilities, and other competing priorities as challenges to acceptance and completion of TPT [31, 33, 34]. As described by Stennis et al., allowing PLHIV to select a mode of delivery for 3HP is likely to increase acceptance and completion [35].

Both PLHIV and health workers were hesitant to promote the use of digital adherence monitoring for PLHIV on SAT since they had no experience in using such technology and therefore perceived it as unreliable for monitoring patient adherence to medicines. The WHO recommends the integration of digital health technologies into TB prevention and care activities in support of its End TB Strateg y[36]. In accordance with this, there is a greater need to empower health workers in resource-limited settings with skills to use this technology for better care. In addition to scale-up of health worker training, promoting confidence in the use of digital technology to monitor patient care can be increased by sharing examples of the successful use of technology elsewhere [37].

The use of the behavioral change wheel implementation framework enabled us to link to several intervention functions that can be utilized to directly address the identified barriers and promote the facilitators. The intervention functions included education, persuasion, environmental restructuring, enablement, and training. For example, as part of the intervention design, we developed a standardized pre-treatment counseling job aid to streamline the information provided to PLHIV before they are initiated on 3HP. In addition, we proposed the establishment of a one-stop point of care for 3HP dosing, refills, and any follow-up to reduce patient waiting time.

In summary, we have used the COM-B model and the corresponding behavior change wheel to identify key facilitators and barriers to the acceptance and completion of 3HP by PLHIV in Kampala, Uganda. Our findings suggest directions for adherence-support interventions, including patient education, enhancing trust, and patient-centered treatment support. These interventions are likely generalizable to other clinical interventions in similar populations in sub-Saharan Africa and other high-burden settings [38, 39].

We are now conducting a randomized trial of different patient-centered treatment support approaches (directly observed therapy, self-administered therapy, or informed choice) to add empirical, quantitative findings to this body of evidence [22].

A major strength of this study is that we interviewed both health care providers and PLHIV; populations which are critical for the scale-up of 3HP-based TB preventive therapy. Importantly, these findings provide a perspective of a wide spectrum of PLHIV who have been in care for varying durations ranging from less than 1 year to 14 years.

Our study has some limitations. First, we collected data at a single health facility which is well resourced for HIV service delivery. The barriers and facilitators may be different from other clinical contexts. Second, although these perspectives were obtained from a wide spectrum of PLHIV receiving care at the clinic, including a balanced gender representation, it is possible that the results may be different with varying cultural contexts. Third, there might be other barriers to 3HP that go beyond the purview of patients and health workers that we were not able to study. However, by combining patient and health worker perspectives of patients, these findings highlight key issues that need to be addressed while considering the scale-up of 3HP-based TPT in high HIV/TB burden settings such as Uganda and beyond.

## Conclusions

Using a formative qualitative and comprehensive theoretical approach, we identified key barriers, facilitators, and appropriate interventions, including patient education, enhancing trust, and patient-centered treatment support that could be used to optimize the delivery of 3HP to PLHIV in our setting. These interventions are likely generalizable to other clinical interventions in similar populations in sub-Saharan Africa and other TB high-burden settings.

## Supplementary Information

**Additional file 1.** Consolidated criteria for reporting qualitative research (COREQ): 32-item checklist.

## Data Availability

The datasets used and/or analyzed during the current study are available from the corresponding author on reasonable request.

## Abbreviations

3HP: Combination of isoniazid and rifapentine medicines taken once-weekly for 12 weeks
PLHIV: Person/people living with HIV
TB: Tuberculosis
HIV: Human immunodeficiency virus
AIDS: Acquired immunodeficiency syndrome
ART: Antiretroviral therapy
ARV: Antiretroviral drug
TPT: Tuberculosis preventive therapy
COM-B: Capability opportunity motivation behavior
BCW: Behavior change wheel framework
SAT: Self-administered therapy
DOT: Directly observed therapy
WHO: World Health Organization
IPT: Isoniazid preventive therapy
SMS: Short message service
